# Laparoscopic resection for a mesenteric lipoma of the ascending colon: a case report

**DOI:** 10.1097/RC9.0000000000000511

**Published:** 2026-05-06

**Authors:** Kei Sugano, Masanori Kotake, Hiroshi Saito, Daisuke Fujimori, Takuo Hara, Noriyuki Inaki

**Affiliations:** a Department of Surgery, Koseiren Takaoka Hospital, Takaoka, Toyama, Japan; b Department of Gastrointestinal Surgery/Breast Surgery, Kanazawa University Hospital, Kanazawa, Ishikawa, Japan

**Keywords:** ascending colon, case report, laparoscopic resection, mesenteric tumor, minimally invasive surgery

## Abstract

**Introduction:**

Lipomas are benign tumors that commonly occur in the arms, legs, and trunk of adults. Mesenteric lipomas are rare, particularly in the mesocolon.

**Case presentation:**

A 32-year-old puerperal woman developed abdominal pain 1 day after a Cesarean section. Computed tomography revealed a 10-cm, poorly enhanced, smoothly contoured mass dorsal to the ascending colon and ventral to the right kidney. Magnetic resonance imaging showed a homogeneous mass with clear borders and a thin peripheral capsule. A retroperitoneal sarcoma and well-differentiated liposarcoma were suspected. The mass was supplied by the superior mesenteric artery; therefore, laparoscopic ileocecal resection was performed. A histopathological examination revealed a 10 × 8 cm mesenteric lipoma. The patient recovered well and was discharged.

**Discussion:**

Mesenteric lipomas are uncommon and mostly arise from mobile intestinal segments, including the small intestine, transverse colon, and sigmoid colon. Since the ascending colon is retroperitoneally fixed, lipomas from this region are exceptionally rare and challenging to diagnose preoperatively. Mesenteric lipomas may mimic retroperitoneal tumors, particularly well-differentiated liposarcomas; therefore, a careful assessment of anatomical relationships and vascular supply is essential. When malignancy cannot be excluded, complete surgical resection allows for diagnostic confirmation and therapeutic management. Laparoscopic surgery provides a minimally invasive approach and safe tumor removal. In this case, laparoscopic ileocecal resection enabled a definitive diagnosis and successful treatment.

**Conclusion:**

Mesenteric lipomas arise from mobile intestinal segments, such as the small intestine, transverse colon, and sigmoid colon. Mesenteric lipomas in the ascending colon have not been described in the English or Japanese literature.

## Introduction

Mesenteric tumors are generally characterized by mobility within the mesentery and attachment to the posterior abdominal wall. Although benign, mesenteric lipomas are rare and have been reported to arise from mobile parts of the intestines, such as the small intestine, transverse colon, and sigmoid colon. The ascending colon is fixed to the retroperitoneum, and to the best of our knowledge, no mesenteric lipomas originating from this region have been reported in the English or Japanese literature.

HIGHLIGHTS

- A mesenteric lipoma arising from the ascending colon is extremely rare and has not yet been reported.
- Differentiation from well-differentiated liposarcomas is essential due to overlapping radiological features.
- Complete laparoscopic resection allowed for a definitive diagnosis and curative treatment.

Furthermore, mesenteric lipomas may be difficult to diagnose. Their radiological features often overlap with those of retroperitoneal tumors, particularly well-differentiated liposarcomas, making a preoperative diagnosis difficult.

The present case is considered to be of educational value, particularly for surgeons and clinicians involved in interpreting abdominal images and diagnosing fatty mesenteric tumors. This case was reported in accordance with the SCARE 2025 criteria^[^1^]^.

## Case presentation

**Figure 1.**
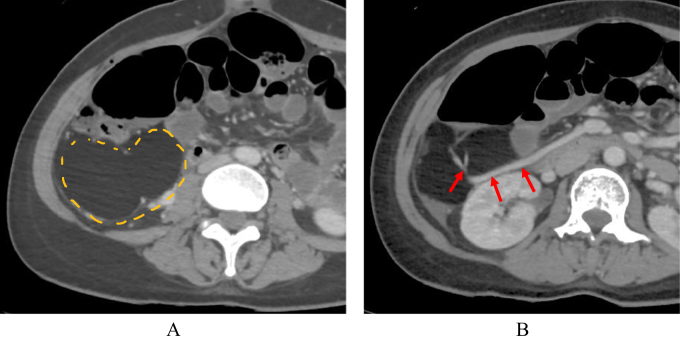
(A) A 10-cm mass behind the ascending colon with a homogeneous internal fat density. (B) The mass was fed by the ileocolic artery and vein (arrows).

A 32-year-old puerperal woman developed abdominal pain one day after a Cesarean section. Her medical history and medication history were unremarkable. There was no family history of intestinal diseases or lipomas. She was a non-smoker and did not consume alcohol. Her height was 160 cm, and her body mass index was 22. The Cesarean incision was clean, without signs of infection. A physical examination showed that her abdomen was flat and soft, with no palpable mass or tenderness. Blood test results were unremarkable, and the levels of the tumor markers, carcinoembryonic antigen and carbohydrate antigen 19-9, were within normal ranges. Computed tomography revealed a 10-cm mass located dorsal to the ascending colon and ventral to the right kidney (Fig. 1a). Magnetic resonance imaging showed a homogeneous, well-circumscribed mass with a thin capsular structure; therefore, a retroperitoneal sarcoma and well-differentiated liposarcoma were suspected. The mass appeared to be supplied by the superior mesenteric artery and vein (Fig. 1b). Based on these findings, laparoscopic ileocecal resection was planned and performed 2 months later.

### Intraoperative findings

**Figure 2.**
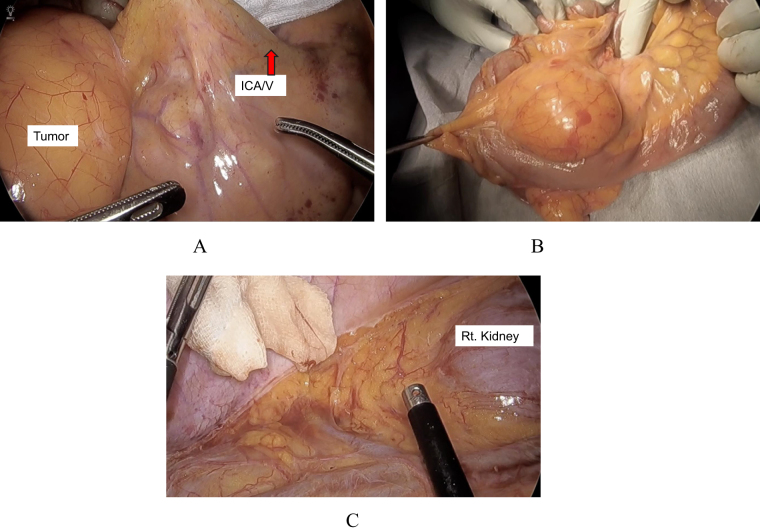
Intraoperative findings. (A) The tumor was located within the mesentery and showed good mobility. It was supplied by the ileocolic artery and vein (arrows). (B) The specimen was extracted extracorporeally without exposing the tumor. (C) Intra-abdominal view after tumor resection.

A 12-mm camera trocar was placed at the umbilicus, and two 5-mm working trocars were inserted into each of the right and left lateral abdominal quadrants. The ileocecal region exhibited good mobility (Fig. 2a). Mobilization of the right colon was performed, including partial resection of the anterior renal fascia (Fig. 2c). The ileocolic vessels were divided. The ileum and ascending colon were transected using a linear stapler. Since it was not possible to exclude the risk of malignancy, resection was performed with adequate surgical margins, without exposing the tumor. Reconstruction was achieved with a functional end-to-end anastomosis. The specimen was extracted by extending the umbilical incision (Fig. 2b).

### Histopathological findings

**Figure 3.**
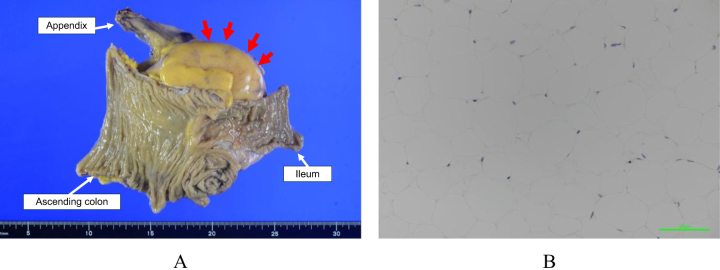
(A) A tumor in the mesentery (arrows) was removed by ileocolic resection. (B) MDM2 IHC (×300) was negative. Mature adipocytes of similar sizes appeared.

A 10 × 8 cm mass was identified within the mesentery. There was no exposure of the intestinal mucosal surface or luminal stenosis (Fig. 3a). Adipocytes were generally uniform in size, and no fibrotic changes were noted. Immunohistochemically, mouse double minute protein 2 (MDM2) was not expressed in proliferating cells, supporting the diagnosis of a benign lipoma (Fig. 3b).

The postoperative course was uneventful, and the patient was discharged on postoperative day six. No signs of recurrence were observed on CT one year after surgery.

## Discussion

Lipomas are benign tumors that commonly arise in the extremities and trunk of adults but rarely in the mesentery. Predisposing factors include obesity, hypercholesterolemia, diabetes, trauma, and a family history of lipomas^[^2^]^. They typically remain asymptomatic due to their slow growth and deep location within the abdominal cavity. However, as they increase in size, they may exert pressure on adjacent structures, leading to a number of symptoms, such as vague abdominal pain, abdominal distension, anorexia, or weight loss^[^3^]^. Fewer than 50 cases of mesenteric lipomas have been reported in the English literature, and all originated from mobile segments of the intestines, such as the small intestine, transverse colon, and sigmoid colon. In this context, the present case is noteworthy because the tumor arose from the mesentery of the ascending colon, a region that is retroperitoneally fixed. To the best of our knowledge, no previous cases involving the ascending colon have been reported in the English or Japanese literature.

The diagnosis of mesenteric lipomas is typically difficult and rarely made preoperatively^[^4^]^. Various differential diagnoses are available for mesenteric tumors. Among these, distinguishing lipomas from liposarcomas is important for therapeutic and prognostic implications. Imaging studies provide some clues: lipomas typically present with a homogeneous internal fat density, smooth margins, and no contrast enhancement. On the other hand, liposarcomas generally have a heterogeneous density, irregular margins, and varying enhancement^[^5^]^. However, difficulties are associated with distinguishing between well-differentiated liposarcomas and lipomas using imaging tests alone. Therefore, a histological examination is useful. Although needle biopsy is applied to definitively diagnose lipomas, mesenteric lipomas have a high risk of vascular and intestinal damage. Therefore, the next option is a surgical biopsy. Furthermore, in symptomatic cases, surgery may be required.

Histopathologically, lipomas and liposarcomas exhibit some differences. They comprise a collection of mature adipocytes and thin fibrous septa. On the other hand, the cell size and chicken wire-like vascular pattern of liposarcomas significantly vary, and they also have thick fibrous septa^[^5^]^. Moreover, liposarcomas frequently exhibit MDM2 gene amplification, which may be detected by immunohistochemistry or fluorescence *in situ* hybridization^[^6^]^. In the present case, the absence of MDM2 expression supported the definitive diagnosis of a benign lipoma.

In the present case, a giant mesenteric lipoma was detected during the puerperal period. Pregnancy and the postpartum period are associated with marked anatomical and physiological changes, and a number of symptoms, such as abdominal pain, nausea, and vomiting, may overlap with pregnancy-related symptoms. Therefore, difficulties are associated with diagnosing an intra-abdominal lipoma during pregnancy^[^7^]^. In the present case, transabdominal and transvaginal ultrasonography had regularly been performed during pregnancy; however, since the patient remained asymptomatic, no intra-abdominal mass was identified.

In addition, female hormones are among the factors that markedly change during pregnancy and the puerperium. Estrogen is an important regulator of adipose tissue metabolism and is known to be involved in lipid metabolism, glucose metabolism, and fat distribution. Estrogen may affect the volume and distribution of adipose tissue through multiple pathways, including lipoprotein lipase, peroxisome proliferator-activated receptor gamma, glucose transporter 4 (GLUT4), and vascular endothelial growth factor (VEGF)^[^8^]^.

However, no consensus has been reached regarding a direct relationship between lipomas and female hormones. Lee *et al* reported that estrogen and progesterone receptors were predominantly negative in an immunohistochemical analysis of soft tissue lipomas, indicating that estrogen and progesterone are unlikely to be major pathogenic factors in lipoma development, whereas frequent VEGF positivity suggested a role for angiogenesis^[^9^]^. On the other hand, subcutaneous lipomas developing after hormone replacement therapy and subcutaneous lipomas enlarging during pregnancy have also been reported^[^10,11^]^. Nevertheless, these studies involved subcutaneous lipomas, and their findings cannot be directly extrapolated to a mesenteric lipoma detected during pregnancy or the puerperal period, as in the present case.

Since lipomas are benign tumors, surgical treatment carries a low risk of malignant transformation. However, it is important to completely remove the fibrous capsule containing the tumor in order to prevent recurrence^[^12^]^. Difficulties are sometimes associated with enucleation, even in benign cases, and the small intestine or colon may need to be resected. In the present case, imaging modalities alone were insufficient for a definitive diagnosis, and a diagnosis was ultimately reached by total resection of the tumor using laparoscopy combined with colon resection. Additionally, surgical resection was required because it was not possible to exclude a potential relationship between the mesenteric lipoma and temporary abdominal pain, and the risk of malignancy remained.

Laparoscopic surgery is a minimally invasive and economically, cosmetically, and physically beneficial method for the treatment of abdominal diseases that has been widely accepted. Complete resection is the standard approach, and since many cases are symptomatic or emergent, laparotomy is often reported. Laparoscopy has also been shown to be useful for treating mesenteric lipomas^[^13^]^. Since the patient was a young puerperal woman, we took advantage of the benefits of laparoscopic surgery, namely, the minimization of pain and a shortened hospital stay, for her and her family.

## Conclusion

We encountered a rare case of a mesenteric lipoma in the ascending colon. Difficulties are often associated with definitively diagnosing lipomas using imaging modalities alone. Although mesenteric tumors may exhibit similar image findings to those of retroperitoneal tumors, the location of the tumor is very important information that impacts the surgical procedure selected and, thus, careful interpretation is required. Laparoscopic surgery, which allows for detailed observations and delicate manipulation, is useful for mesenteric lipomas.

## Data Availability

The datasets supporting the conclusions of this article are included within the article.
